# A Host Defense Mechanism Involving CFTR-Mediated Bicarbonate Secretion in Bacterial Prostatitis

**DOI:** 10.1371/journal.pone.0015255

**Published:** 2010-12-07

**Authors:** Chen Xie, Xiaoxiao Tang, Wenming Xu, Ruiying Diao, Zhiming Cai, Hsiao Chang Chan

**Affiliations:** 1 Epithelial Cell Biology Research Center, School of Biomedical Sciences, Faculty of Medicine, Chinese University of Hong Kong, Hong Kong, China; 2 The Chinese University of Hong Kong Joint Laboratory of Reproductive Medicine, Sichuan University, West China Second University Hospital, Hong Kong, China; 3 Guangdong Key Laboratory of Male Reproduction and Genetics, Peking University Shenzhen Hospital, Shenzhen, China; Sun Yat-Sen University, China

## Abstract

**Background:**

Prostatitis is associated with a characteristic increase in prostatic fluid pH; however, the underlying mechanism and its physiological significance have not been elucidated.

**Methodology/Principal Findings:**

In this study a primary culture of rat prostatic epithelial cells and a rat prostatitis model were used. Here we reported the involvement of CFTR, a cAMP-activated anion channel conducting both Cl^−^ and HCO_3_^−^, in mediating prostate HCO_3_^−^ secretion and its possible role in bacterial killing. Upon *Escherichia coli* (*E coli*)-LPS challenge, the expression of CFTR and carbonic anhydrase II (CA II), along with several pro-inflammatory cytokines was up-regulated in the primary culture of rat prostate epithelial cells. Inhibiting CFTR function *in vitro* or *in vivo* resulted in reduced bacterial killing by prostate epithelial cells or the prostate. High HCO_3_^−^ content (>50 mM), rather than alkaline pH, was found to be responsible for bacterial killing. The direct action of HCO_3_^−^ on bacterial killing was confirmed by its ability to increase cAMP production and suppress bacterial initiation factors in *E coli*. The relevance of the CFTR-mediated HCO_3_^−^ secretion in humans was demonstrated by the upregulated expression of CFTR and CAII in human prostatitis tissues.

**Conclusions/Significance:**

The CFTR and its mediated HCO_3_^−^ secretion may be up-regulated in prostatitis as a host defense mechanism.

## Introduction

Prostatitis is one the most common urological disorders in men and up to half of the male population may suffer from prostatitis at some time in their lives [1]. Prostatitis usually presents with irritative or obstructive voiding symptoms, genitourinary, pelvic or rectal pain, and is sometimes associated with sexual dysfunction and infertility [2], [3], [4], [5], [6]. According to the recent consensus definition by the National Institute of Health (NIH), prostatitis can be classified into 4 categories. The first 2 categories include prostatitis with bacterial infection, acute bacterial prostatitis (Category I) and chronic bacterial prostatitis (Category II). Chronic nonbacterial prostatitis/chronic pelvic pain syndrome belongs to category III and asymptomatic inflammatory prostatitis belongs to category IV. Clinically, only 5–10% of the prostatitis cases are diagnosed with bacterial infection (category I and II), of which 50–80% is caused by *E coli* [7], while nonbacterial prostatitis accounts for more than 90–95% of the clinical cases [1], [8]. Irregardless the types of prostatitis, with bacterial infection or not, the common feature in most cases is the inflammation of the prostate gland with the presence of white blood cells or elevated levels of cytokines, especially IL-1β and TNF-α, in the expressed prostate secretion (EPS) or post-prostate-message urine [9], [10], [11].

Another hallmark of prostatitis is an alkaline shift in pH consistently found in the expressed prostate secretion (EPS), which appears to accompany with the inflammatory response of the prostate with or without bacterial infection[2], [12], [13], [14], [15], [16], [17]. The pH value of the prostate fluid appears to reflect the intensity of inflammation reaction and in general, the more serious inflammation as reflected by larger number of white blood cells, the more alkaline of the pH value [16], [18]. It has also been reported that the pH of EPS in bacterial prostatitis is significantly higher than that in nonbacterial prostatitis [19]. While the marked increase in the pH of EPS has been considered of diagnostic value [14], [16], [17], it is also thought to be one of the reasons for poor results of antibiotic therapy [15]. Normal human prostatic fluid has a pH value between 6.2–6.6, which is significantly lower than that of the plasma value of 7.4 [14], [17]. This pH gradient allows electrically neutral molecules, e.g. drugs and antibiotics, to penetrate into the prostate, become ionized and be trapped or concentrated in the prostate fluid [2], [20], [21]. However, upon inflammation, the prostate fluid may become markedly alkaline (> pH 8.0), which may affect the concentration of drugs or antibiotics in the prostate [14]. The variation in pH in prostatitis may also considerably alter the therapeutic efficacy of antibiotics, apart from their reduced concentrations in the prostate [2].

Despite the diagnostic and therapeutic implications of the pH in the EPS, the molecular mechanism governing the pH regulation of the prostate fluid in normal and inflammatory state remains large unknown. The glandular epithelium of the prostate is known to secrete citric acid, which is thought to maintain the osmotic pressure and pH of the prostate fluid. The pH increase observed in prostatitis has been proposed to be due to impaired secretory function of the prostate (i.e. reduction in citric acid level) upon inflammation [17]. However, whether the increase in pH seen in prostatitis is simply due to a decrease in the relative level of citric acid, or an increase in the secretion of alkaline substances or ions, such as bicarbonate, is not clear. More importantly, the question as to whether the characteristic increase in prostatic fluid pH in prostatitis is of any physiological significance has not been addressed.

The cystic fibrosis transmembrane conductance regulator (CFTR) is a cAMP-activated ion channel which is found in a wide variety of epithelial tissues including the lung, liver, pancreas, intestine, reproductive tracts and sweat glands [22], [23], [24], [25]. Mutations in the CFTR gene are known to cause cystic fibrosis (CF), a lethal genetic disease found among Caucasian, which is characterized by defective Cl^−^ and HCO_3_^−^ secretion [26]. CFTR may conduct HCO_3_^−^ directly as an anion channel with measured HCO_3_^−^ permeability [27] or indirectly as a Cl^−^ channel working in parallel with Cl^−^/HCO_3_^−^ exchangers to provide a recycling pathway[28], [29]. In fact, our previous studies have demonstrated the involvement of CFTR in mediating uterine and oviductal HCO_3_^−^ secretion, which are vital to the fertilizing capacity of sperm and embryo development [30], [31], [32]. Since CFTR is also known to be expressed in the human prostate [33], [34], [35], it may also play a role in prostatic HCO_3_^−^ secretion as well although the physiological role of CFTR in the prostate has not been elucidated.

It has been reported that under the circumstances of inflammation, or upon bacterial infection, the increased release of inflammatory cytokines such as IL-1β and TNF-α have potent effect on up-regulation of CFTR in epithelial cells [36], [37]. Since increased levels of inflammatory cytokines including IL-1β and TNF-α are also found in prostatitis, with or without bacterial infection, they may also up regulate CFTR in the prostate, thereby enhancing HCO_3_^−^ secretion and leading to the characteristic pH increase in prostatitis. Interestingly, recent studies have indicated the possible involvement of defective CFTR-mediated HCO_3_^−^ secretion in the pathogenesis of CF [30], [38], [39]. There seems to be a link between defective HCO_3_^−^ secretion and higher risk of infection in CF. Most CF patients, about 95%, die from lung infection with airway acidification found [40]. It has been reported that the acidity in CF airways may be due to defective HCO_3_^−^ ion transport [41], although the exactly role of HCO_3_^−^ in CF pathogenesis is still not fully understood. Of note, HCO_3_^−^ has been implicated in bacterial killing, however whether its action is direct or indirect remains unclear [42], [43], [44], [45]. Recently it has been reported that in the presence of carbonate, the susceptibility of bacteria to antimicrobial peptides may be increased since carbonate may induce global changes in the structure and gene expression of bacteria [46]. Thus, the increased pH observed in prostatitis would be of physiological significance for host defense against bacterial infection and the pH increase should be due to enhanced prostatic HCO_3_^−^ secretion.

Taken together, we hypothesized that CFTR might be involved in prostatic HCO_3_^−^ secretion, its upregulation by inflammatory cytokines and thus enhanced HCO_3_^−^ secretion might be responsible for the hallmark increase in pH observed in prostatitis. We further hypothesized that the enhanced CFTR-mediated HCO_3_^−^ secretion in prostatitis might be an important host defense mechanism of the prostate against bacterial infection. We undertook the present study to test these hypotheses using a primary culture of rat prostatic epithelial cells and a rat prostatitis model. We demonstrated the expression of CFTR in rat prostatic epithelium and its involvement in prostatic bicarbonate secretion. The results show that CFTR as well as carbonic anhydrase II (CAII), which is a key enzyme responsible for conversion of HCO_3_^−^ from CO_2_, could be up-regulated during prostate inflammation in the animal model and human prostatic tissues with inflammation. The role of CFTR in host defense of the prostate was demonstrated by impaired bacterial killing activity upon interfering with CFTR function *in vitro* and *in vivo*. The direct effect of HCO_3_^−^ on bacterial killing and possible underlying mechanism were also investigated. The present results have provided the molecular mechanism underlying the long observed pH increase in prostatitis and demonstrated a previously unsuspected role of CFTR in the host defense of the prostate.

## Results

### CFTR expression in the epithelial cells of rat ventral prostate

**Figure 1:**
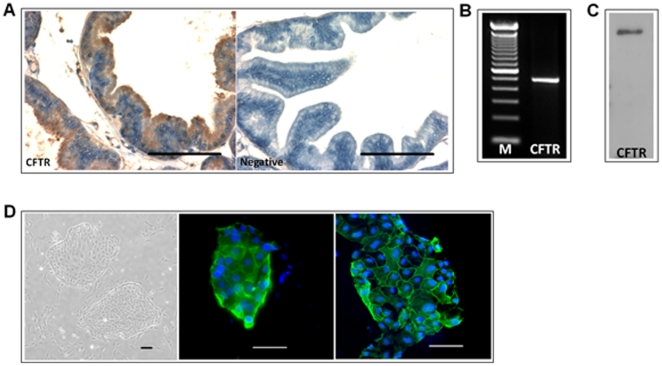
Expression of CFTR in rat prostate epithelial cells. (A) Immunohistochemical staining of CFTR in SD rat prostate with negative control in the absence of primary antibody. CFTR was expressed at the apical surface of rat ventral prostate epithelium. Scale bar: 100 µm. (B) CFTR transcript was detected by RT-PCR in cultured rat prostate epithelial cells with predicted amplification products at 481 bp. (C) CFTR protein was detected in rat prostate epithelial cells by Western blotting which recognizes a band at MW 160 kDa. (D) Phase contrast image (left) and immunofluorescence staining of cytokeratin 5&8 (middle, green) or CFTR (right, green) in rat prostate epithelial cells. Cell nuclei were counterstained with DAPI (blue). Scale bar: 100 µm.

In order to investigate the role of CFTR in prostatitis, we first examined its expression in rat prostate since CFTR expression in the prostate has not been demonstrated in any species other than the human. Immunohistochemistry revealed that CFTR immunoreactivity was detected in the apical surface of the epithelial cells of the rat ventral prostate (Figure 1A). We further confirmed CFTR expression in a primary culture of rat prostate epithelial cells. RT-PCR results revealed a PCR product with expected size of rat CFTR (481 bp) (Figure 1B). Western blot also showed a band of 160 KD as expected of CFTR (Figure 1C). Immunofluorescent staining also localized CFTR protein to the plasma membrane of the culture epithelial cells (Figure 1D, right), which was also stained positive for cytokeratin 5&8, a marker of epithelial cells (Figure 1D, middle).

### Involvement of CFTR in mediating prostatic bicarbonate secretion

**Figure 2:**
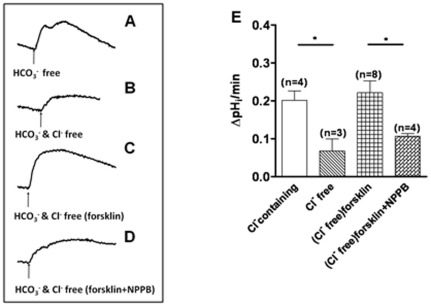
Involvement of CFTR in mediating cAMP-stimulated bicarbonate secretion by rat prostate epithelial cells. (A) The pH_i_ recovered quickly after cellular alkalinization induced by removing bicarbonate/CO_2_ from perfusate in the presence of Cl^−^. (B) The rate of pH_i_ recovery was markedly attenuated when extracellular Cl^−^ was removed from the perfusate. (C) In the absence of Cl^−^, forskolin (forsk, 10 µM) stimulated pH_i_ recovery. (D) The forskolin-induced pHi recovery could be blocked by NPPB (100 µM). The scales in A–D are the same. (E) Summary of pH_i_ recovery rates under different conditions after cellular alkalinization induced by removing bicarbonate/CO_2_ from perfusate. (*P<0.05).

To investigate the role of CFTR in prostatic HCO_3_^−^ secretion, we measured the intracellular pH (pH_i_) in the established culture of prostate epithelial cells. Cellular alkalization was induced by removing HCO_3_^−^/CO_2_ from the perfusate (Figure 2A), and the rate of pH_i_ recovery, which reflects HCO_3_^−^ extrusion, was measured. When extracellular Cl^−^ was removed from the apical perfusion solution, the rate of pHi recovery was greatly attenuated in comparison with that in the Cl^−^-containing solution (Figure 2B), indicating the operation of a Cl^−^-dependent HCO_3_^−^ extrusion process, probably involving an anion exchanger. However, the rate of pH_i_ recovery, in the absence of Cl^−^ or inactivation of the Cl^−^/HCO_3_^−^ exchanger, could be increased by an adenylyl cyclase activator, forskolin, indicating a cAMP-dependent HCO_3_^−^ extrusion pathway (Figure 2C). The forskolin-induced pH_i_ recovery could be blocked by NPPB (100 µM) (Figure 2D), a blocker known to inhibit CFTR, suggesting that CFTR may be responsible for the HCO_3_^−^ secretion in the cultured prostate epithelial cells. These results indicate a direct role of CFTR in mediating prostate HCO_3_^−^ secretion and an indirect role, possibly working in parallel with a Cl^−^/HCO_3_^−^ exchanger for HCO_3_^−^ extrusion.

### Rat prostate epithelial cells respond to *E coli*-LPS with up-regulated cytokines, CFTR and CA II expression

**Figure 3:**
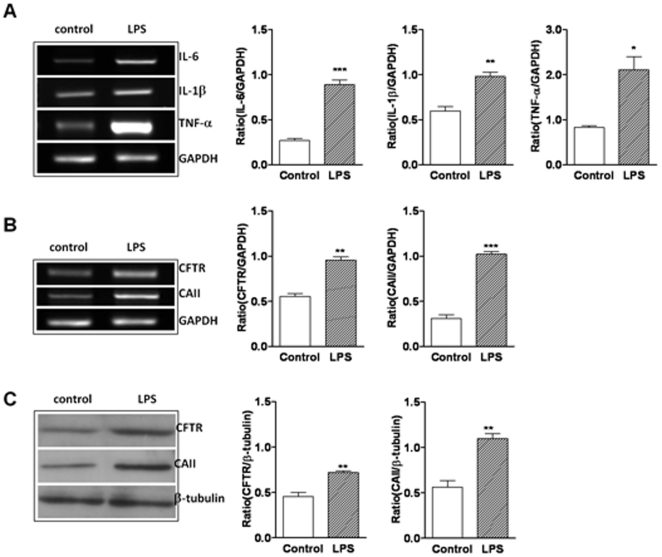
LPS-induced upregulation of cytokines, CFTR and CAII expression in rat prostate epithelial cells. Primary rat prostate epithelial cells were treated with 1 µg/ml *E.coli*-LPS for 24 h. The expression levels of IL-6, IL-1β, TNF-α, CFTR and CAII were evaluated by RT-PCR (A, B) and GAPDH was used as control. Data were from three experiments. (C) *E.coli*-LPS up-regulated the protein expression of CFTR (160 kD) and CAII (29 kD)as detected by western blot, with β-tubulin (55 kD) used as the loading control. Data were from three experiments. (*P<0.05, **P<0.01, ***P<0.001).

To mimic bacteria-induced inflammation in prostatitis, *Ecoli*-LPS, an endotoxin present in the outer membrane of the bacteria was used. We challenged the cultured prostatic epithelial cells with1 ug/ml *E.coli*-LPS for 24 h and performed RT-PCR to examine the expression of pro-inflammatory cytokine IL-6 (414 bp), IL-1β (313 bp) and TNF-α (292 bp), with GAPDH (340 bp) as the internal marker. We observed that IL-6, IL-1β and TNF-α mRNA expression were upregulated by *E.coli*-LPS (Figure 3A). We also examined the effect of *E.coli*-LPS on CFTR and CAII expression in cultured prostate epithelial cells. As shown in Figure 3B and 3C, mRNA and protein expression of CFTR and CAII were significantly up-regulated upon stimulation of *E.coli*-LPS, respectively. These results suggest that CFTR and its mediated HCO_3_^−^ secretion may be up-regulated by inflammatory cytokines upon bacterial infection/inflammation, resulting in the alkaline shift in pH observed in prostatitis.

### Bactericidal capacity of prostatic epithelial cells *in vitro* and involvement of CFTR

**Figure 4:**
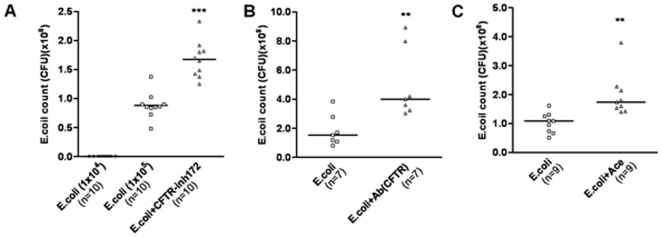
Involvement of CFTR and CAII in bacterial killing *in vitro*. (A)When 1×10^4^ CFU of *E.coli* was inoculated to the apical compartment of the rat prostate epithelial cells for 18 h, there was no bacterial activity detected in the culture medium. 10 µM CFTR_inh_-172 (A), 1∶500 CFTR antibody (B) or 50 µM acetazolamide (C) were added with 1×10^5^ *E.coli* to block CFTR or CAII activity and their effect on bacterial activity 18 hours after incubation was shown. (**P<0.01, ***P<0.001).

What is the physiological significance of the upregulation of CFTR and the possible enhanced CFTR-mediated prostatic HCO_3_^−^ secretion during prostatitis? Since HCO_3_^−^ has been implicated in bacterial killing, the enhanced CFTR-mediated HCO_3_^−^ secretion in prostatitis may serve as a host defense mechanism. To test this, we inoculated 1×10^4^ colony-forming units (CFU) of the gram-negative bacteria *E.coli*, which are responsible for up to 80% of the bacterial prostatitis in humans, to the primary culture of rat prostate epithelial cells and found no bacterial colony growth in the collected medium, indicating bactericidal capacity of the epithelial cells. When 1×10^5^ CFU *E*.*coli* were added to the cells in the absence or presence of CFTR inhibitor, CFTR_inh_-172 (10 µM) or CFTR antibody, we observed significantly larger number of bacterial colonies in the medium collected from the CFTR_inh_-172 and CFTR antibody treatment groups as compared to the non-treated control group (Figure 4A, B). To test whether this bactericidal activity was due to HCO_3_^−^, the cells were pretreated with carbonic anhydrase inhibitor, acetazolamide (50 µM), and it was found that the bactericidal capacity of the culture was greatly attenuated (Figure 4C). As controls, direct addition of CFTR inhibitor/antibody or acetazolamide to the bacterial culture (1×10^5^ CFU) at the concentrations used did not affect the growth of *E.coli*, excluding direct effect of the inhibitors and antibody on the bacteria.

### Involvement of CFTR in bacterial killing *in vivo*

**Figure 5:**
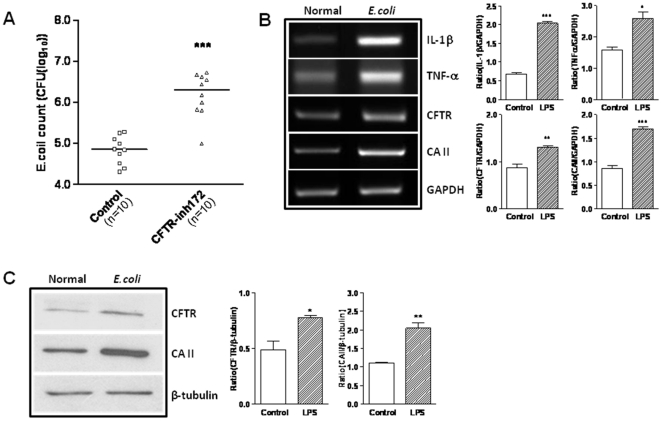
Bacterial killing effect of CFTR *in vivo* and upregulation of cytokines, CFTR and CAII in *E coli*-infected rat prostate. (A) Comparison of *E coli* bacterial activities recovered from rat prostatitis models without or with CFTR_inh_-172 (10 µM). Each point indicates the bacterial CFU per gram of prostate tissue weight (***P<0.001). (B) *E.coli* up-regulated the expression of cytokine genes, CFTR and CAII in rat prostate as determined by RT-PCR. Data were from three experiments. (C) Expression of CFTR (160 kD) and CAII (29 kD) protein was significantly up-regulated in *E.coli* -infected rat prostate as determined by western blot. Data were from three experiments. (*P<0.05, **P<0.01, ***P<0.001).

To further confirm the involvement of CFTR-mediated HCO_3_^−^ secretion in bacterial killing in a physiological context, we established an *in vivo* prostatic bacterial infection model. Following bacterial inoculation (2×10^7^ CFU) to the prostate, all samples collected from the bacterial prostatitis groups showed acute inflammation with severe infiltration of polymorphonuclear cells (PMNs) into bacteria-containing space. Injecting *E.coli* combined with 10 uM CFTR_inh_-172 resulted in a significantly higher number of bacteria isolated from the prostates, as compared to the control, 6.422±0.168 log_10_ CFU/g vs. 5.224±0.102 log_10_ CFU/g, respectively (Figure 5A). We further examined the expression of CFTR, CAII, IL-1β and TNF-α and they were all up regulated in bacteria-infected prostates (Figure 5B, C). These results suggest that the CFTR-mediated HCO_3_^−^ secretion may be up-regulated upon bacterial infection and involved in bacterial killing *in vivo*.

### Bicarbonate itself but not alkaline pH is mainly responsible for bacterial killing

**Figure 6:**
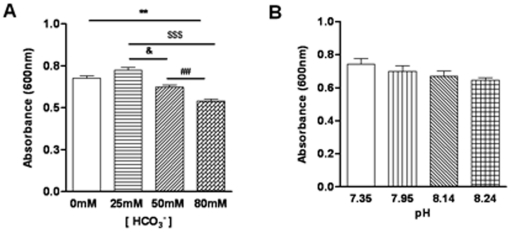
HCO_3_^−^ but not pH exhibits bactericidal capacity *in vitro*. (A). The activity of *E.coli* was inhibited by 80 mM HCO_3_^−^ and 50 mM HCO_3_^−^. (B) Insignificant effect on bacterial activities of varied pH (at constant 25 mM HCO_3_^−^) at 7.35, 7.95, 8.14 and 8.24 which was corresponding with the pH value of different concentration of HCO_3_^−^. Data were from three experiments. (**P<0.01vs 0 mM HCO_3_^−^, ^$$$^p<0.001 vs 25 mM HCO_3_^−^,^ ##^P<0.01vs 50 mM HCO_3_^−^, ^&^P<0.05 vs 25 mM HCO_3_^−^).

Since HCO_3_^−^ is weak base and the bactericidal effect we observed *in vitro* and *in vivo* could be due to its buffering effect on the pH rather than HCO_3_^−^ itself. To distinguish the two, we tested the bactericidal capacity of solutions with different concentrations of HCO_3_^−^. Our results showed that the bacterial count of *E.coli* was significantly reduced by 80 mM HCO_3_^−^ with a significantly lower absorbance ratio as compared to other lower HCO_3_^−^ concentrations tested (Figure 6A). To exclude possible involvement of pH in the bacterial killing, we made up solutions with different pH (7.35–8.24) while keeping a constant HCO_3_^−^ concentration at 25 mM. The results showed that there was no significant difference in the bacterial killing capacity between groups with different pH values (Figure 6B). These results suggested that the antibacterial activities observed with 80 mM or 50 mM HCO_3_^−^ were mainly due to HCO_3_^−^ itself but not alkaline pH.

### Bicarbonate increases intracellular cAMP of *E.coli*

**Figure 7:**
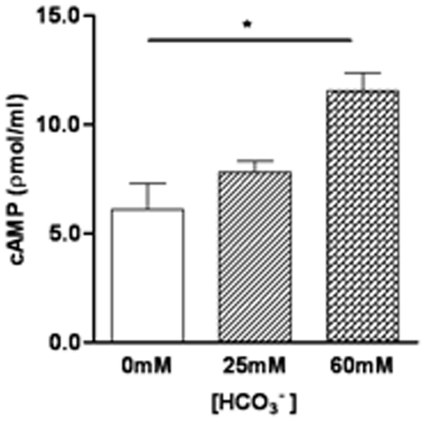
Concentration-dependent effect of HCO_3_^−^ on intracellular cAMP production in *E.coli*. The intracellular cAMP concentration in *E.coli* treated with 60 mM HCO_3_^−^ was significantly higher than that with 0 mM or 25 mM HCO_3_^−^ (*P<0.05). Data were from three experiments.

What might be the molecular mechanism underlying the bacterial killing effect of HCO_3_^−^? Since elevated cAMP levels in bacteria are known to suppress protein synthesis [47], [48] and HCO_3_^−^ is known to activate a soluble form of adenylate cyclase (sAC) [49], we tested whether HCO_3_^−^ exposure could result in elevation of cAMP in bacteria. When *E.coli* were exposed to different concentrations of HCO_3_^−^, their intracellular cAMP levels were elevated in a HCO_3_^−^ concentration-dependent manner (Figure 7), as measured by the Elisa kit.

### Bicarbonate suppresses the expression of initiation factors IF1, IF2 and IF3 in *E.coli*

**Figure 8:**
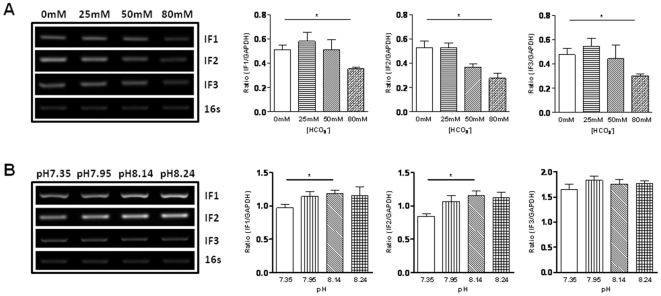
Effect of HCO_3_^−^ and pH on expression of *E. coli* initiation factors IF1, IF2 and IF3. (A) The mRNA expression of initiation factors was significantly inhibited by 80 mM HCO_3_^−^. (B) Alkaline pH with a constant HCO_3_^−^ of 25 mM did not suppress IF1, IF2 and IF3 gene expression. 16 s was used as control. Data were from three experiments. (*P<0.05).

To further confirm a direct bactericidal activity of HCO_3_^−^, its effect on suppressing the genes of bacterial initiation factors was examined. As shown in Figure 8, RT-PCR analysis of *E.coli* cultured in the absence or different concentrations of NaHCO_3_ showed that the expression of IF1, IF2 and IF3 genes was significantly inhibited by HCO_3_^−^ at the concentration of 80 mM as compared to HCO_3_^−^ at 0 mM. However, alkaline pH with a constant HCO_3_^−^ of 25 mM did not suppress these genes expression, excluding significant involvement of pH in bacterial killing.

### The expression of CFTR and CAII is up-regulated in human prostatitis

**Figure 9:**
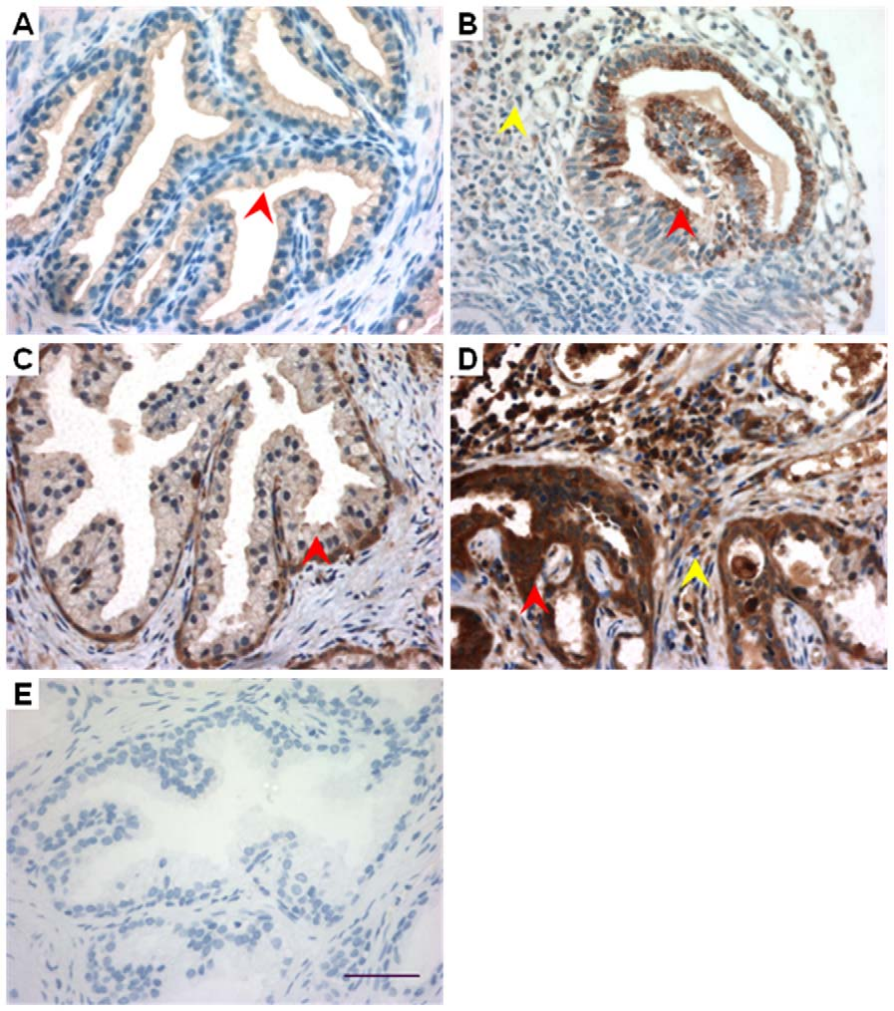
Expression of CFTR and CAII in human hyperplasia prostate with inflammation. CFTR (A, B) and CAII (C, D) were detected in human hyperplasia tissues. There was lymphocytes infiltration (Yellow arrow, B, D) in the inflamed area of the clinical prostate hyperplasia samples. Note that the expression of CFTR and CAII was stronger in the area with lymphocytes infiltration (Red arrow, B, D) than those without infiltration (Red arrow, A, C). (E) Negative control. Scale bar: 50 µm.

While both *in vitro* and *in vivo* experiments on rat prostate epithelial cells strongly indicated that the CFTR-mediated HCO_3_^−^ secretion is enhanced in prostatitis, which has bactericidal capacity, it remains to be confirmed that this host defense mechanism is also present in human prostate. We thus examined CFTR and CAII expression in human prostate hyperplasia samples with inflammation. Immunohistochemical results showed CFTR immunoactive signal at the apical border of the human prostatic epithelium (Figure 9A). Compared to the tissue without inflammation, the expression of CFTR in the prostate glands evident of lymphocytes infiltration (Figure 9B) was much stronger, indicating that the expression of CFTR was up-regulated in human prostatitis. The expression of CAII was detected in the cytoplasm of human prostate epithelial cells (Figure 9C), which was also up-regulated in the inflamed area of the clinical prostate hyperplasia samples (Figure 9D). The upregulation of both CFTR and CAII in the inflamed human prostate tissues indicate that the CFTR-mediated HCO_3_^−^ secretion may also be enhanced upon inflammation, which may be responsible for the high pH observed in human prostatitis.

## Discussion

Almost all cases of prostatitis, whether it is bacterial or non-bacterial, exhibit a characteristic alkaline shift in pH [12], [13], [14], [15], [16]; however, the question as to how this change in pH is brought about in prostatitis has not been addressed to any significant extent. The present study has investigated the possible mechanism underlying this pH change and demonstrated for the first time the involvement of CFTR in mediating prostatic HCO_3_^−^ secretion, which may be enhanced upon bacterial infection or inflammation. Apart from demonstrating CFTR expression in rat prostatic epithelium, which is consistent with that previously found in human prostate [33], [34], [35], we have also elucidated the functional roles of CFTR in the prostate in health and disease. By measuring the rate of pHi recovery from cellular alkalization, in the presence or absence of extracellular Cl^−^ in conjunction with the use of the cAMP agonist and CFTR inhibitor, we have demonstrated that prostatic epithelium can extrude HCO_3_^−^ under unstimulated condition through a Cl^−^/HCO_3_^−^ exchanger since removal of extracellular Cl^−^ greatly attenuated the pHi recovery rate. Under this condition, when the anion exchanger is inactivated, prostatic epithelium can be stimulated to secrete HCO_3_^−^ through a cAMP-dependent pathway which can be blocked by CFTR inhibitor. Therefore, similar to what we previously found in the uterus[30] and oviduct [32], CFTR in the prostate appears to be involved in HCO_3_^−^ secretion either directly or indirectly as a recycling pathway for Cl^−^ to facilitate the operation of the anion exchanger, although the identity of the exchanger remains to be elucidated. Of note, the expression of SLC26A3 in the human prostate has been reported [50] and we have also detected SLC26A6 in rat prostate (Xie C unpublished data), both of which are known to be able to transport HCO_3_^−^ across the apical membrane of many epithelia [51] and work in concert with CFTR. The CFTR-mediated prostatic HCO_3_^−^ secretion may be important for sperm motility upon ejaculation since prostate secretion is known to contribute to the semen volume and HCO_3_^−^ is the key factor triggering sperm motility [52], [53]. The physiological role of the CFTR-mediated HCO_3_^−^ secretion under normal condition warrants further studies.

One important observation made in this study is the upregulation of CFTR and CAII, along with several important inflammatory cytokines, upon *E. coli-*LPS challenge *in vitro* or *E. coli* infection *in vivo*. This suggests that a physiological consequence to bacterial infection or LPS challenge in the prostate would be the enhancement of the CFTR-mediated HCO_3_^−^ secretion, which may be responsible for the increase in pH observed in prostatitis. Cytokines, such as IL-1β and TNF-α, have been reported to upregulate CFTR expression [36], [37], [54]. IL-1β could activate the NF-κB protein, enabling it to enter the nucleus and bind to the κB-like response element at position -1103 to -1093 in the CFTR 5′-regulatiory element with a subsequent increase in CFTR promoter activity, resulting in increased CFTR mRNA [55]. The observed upregulation of CFTR and CAII upon bacterial infection or LPS challenge could be due to the elevated levels of cytokines induced by the pathogens. Since both bacterial and non-bacterial prostatitis have elevated levels of cytokines, including IL-1β and TNF-α [9], [10], these cytokines may enhance HCO_3_^−^ production and HCO_3_^−^ transport by upregulating CAII and CFTR. This may explain the characteristic alkaline shift in pH in almost all categories of prostatitis. Of clinical interest, it has long been observed in prostatitis the more severe the inflammation (e.g. the larger number of infiltrated PMNs), the greater the alkaline shift in pH found [16]. Interestingly, the present study also found enhanced CFTR and CAII expression in human hyperplasia tissues where inflammation was more prominent. Taken together, the present study has provided a molecular mechanism underlying prostatic HCO_3_^−^ secretion and its upregulation in bacterial infection or upon inflammation, which may explain the long observed pH increase associated with prostatitis.

More importantly, the present study has also demonstrated the physiological significance of the CFTR-mediated prostatic HCO_3_^−^ secretion in the host defense against bacterial infection. While the characteristic increase in pH in prostatitis has been considered of diagnostic value [18], its physiological role has not been explored. In this study, both *in vitro* and *in vivo* experiments have demonstrated that prostatic epithelial cells have bactericidal activities, which are largely dependent on CFTR-mediated HCO_3_^−^ secretion since inhibitor of either CFTR or CAII greatly attenuates bacterial killing by both the primary prostatic epithelial culture and rat prostate. Since CFTR is involved in prostatic HCO_3_^−^ secretion, the CFTR-dependent bacterial killing capacity of the prostate could be due to a direct effect of HCO_3_^−^ or indirect effect through altered pH. We have demonstrated that the HCO_3_^−^, rather than alkaline pH, is mainly responsible for bacterial killing since varying pH at a constant HCO_3_^−^ concentration does not produce significant bactericidal effect, whereas, significant bacterial killing can be observed at a HCO_3_^−^ concentration greater than 50 mM. One immediate question that follows is whether the prostate can secrete such high concentrations of HCO_3_^−^. The answer is yes since the mean pH often observed in prostatitis is 8–8.3 [56], which, according to Henderon-Hasselbalch equation, is equivalent to over 90 mM HCO_3_^−^. This high concentration of HCO_3_^−^, as demonstrated in the present study, is able to significantly reduce bacterial activity. We have further demonstrated that HCO_3_^−^ does so by elevating bacterial cAMP levels, presumably through its well-established sensor soluble adenylyl cyclase [49], thereby suppressing the bacterial initiation factors genes, which are known to participate in the process of protein biosynthesis in *E.*coli [57], [58]. IF1 could stimulate IF2 and IF3 activities and act as a protein factor for the stabilization of the initiation complex that is essential for cell viability [59]. The observed suppressing effect of HCO_3_^−^ on these initiation factors is consistent with its bactericidal activity.

The bactericidal effect of HCO_3_^−^ could be explained by its presently demonstrated ability to induce an increase in cAMP in the bacteria since activation of cAMP has been reported to suppresse protein synthesis in bacteria [48]. Of note, the expression of sAC, a distinctive form of adenylyl cyclase, in bacteria is well documented [60], [61] and its role as a HCO_3_^−^ sensor has been reported to induce cAMP increase in a number of cell types including lung, sperm [62], [63]. Taken together, HCO_3_^−^ may exert its bactericidal effect by acting on sAC to induce increase in cAMP production, which in turn suppresses protein synthesis and thus reduces the viability of bacteria. While most previous studies have implicated HCO_3_^−^ in bacterial killing either by altering pH [64] or increase the susceptibility of bacteria to antimicrobial peptides [46]. In the present study, we have demonstrated a direct bacterial killing effect of HCO_3_^−^ through cAMP dependent pathway. Together with the demonstrated CFTR and CAII upregulation, therefore enhanced prostatic HCO_3_^−^ secretion, upon bacterial infection, the present finding suggests a host defense mechanism against bacterial infection in the prostate. The long observed alkaline shift in pH in prostatitis turns out to be physiologically important. Of note, it has been reported that the pH in bacterial prostatitis is significantly higher than the nonbacterial prostatitis [19]. In fact, this could be the reason why a surprisingly low prevalence of bacterial prostatitis, only 5–10%, is found among all cases of prostatitis [65]. We suspect that considerable number of cases of bacterial infection would be gone unnoticed because of the enhanced CFTR-mediated HCO_3_^−^ secretion upon bacterial infection. Consistent with this notion, the present study found that when low CFU of *E coli* was inoculated to the prostatic cell culture, no bacterial activity was found after 18 hours incubation, indicating the bactericidal capacity of the prostatic epithelial cells. However, this bactericidal capacity is greatly attenuated by treatment with CFTR inhibitor or antibody. Taken together, the present finding has revealed a previously undefined role of CFTR and its mediated prostatic HCO_3_^−^ secretion in the host defense against bacterial infection. The present findings may have implications beyond prostatitis since CFTR is expressed in a wide variety of tissues where bacterial infections are readily contracted. For example, patients with cystic fibrosis, a genetic disease caused by CFTR mutations, frequently present with chronic lung infection but the exact cause remains obscure. In light of the present finding, this may now be explained by possible defect in the secretion of bacterial killing HCO_3_^−^ due to CFTR mutations.

Although the present findings provide an explanation to the characteristic increase in pH in both bacterial and non-bacterial prostatitis, we have only elucidated its importance in bacterial prostatitis. It remains unclear whether the elevated HCO_3_^−^ content or alkaline pH would be of any physiological significance in non-bacterial prostatitis, the most common and least understood form of prostatitis. Of note, it has been reported that cAMP is a key intracellular second messenger, which at increased levels has been shown to have anti-inflammatory and tissue-protective effects [66]. An increase in cAMP level during inflammation has been shown to inhibit the proinflammatory and tissue-destructive properties of leukocytes [67]. It is therefore tempting to speculate that the enhanced CFTR-mediated prostatic HCO_3_^−^ secretion is not only important for bacterial killing in bacterial prostatitis, but may also play a key role in non-bacterial prostatitis by increasing the cAMP level of immune cells to suppress their inflammatory responses. Interestingly, extracellular pH has also been suggested to play a role in modulating inflammation. For example, neutrophils have been reported to be less active at a pH of 7.4 than at a lower pH [68]. Therefore, both elevated HCO_3_^−^ content or alkaline pH (as compared to a normal pH of 6.3 in the prostate) observed in non-bacterial prostatitis may be important for suppressing inflammation. Further work is required to investigate these possibilities.

In summary, the present study has elucidated the molecular mechanism underlying the long observed but unexplained characteristic alkaline shift in pH in prostatitis and revealed a previously undefined role of CFTR in host defense against bacterial infection in the prostate. The present findings also point to the possible role of the CFTR-mediated HCO_3_^−^ secretion in anti-inflammatory process. Further work along this line will not only confirm the diagnostic value of the characteristic pH increase in prostatitis but may also provide new strategies for the treatment of prostatitis.

## Materials and Methods

### Animals

Male 4-week-old and 12-week-old SD rats were kept in the Laboratory Animal Service Center of the Chinese University of Hong Kong. Ethics committee approval was obtained prior to the study (Approval ID: 07/090/MIS) and the animal experiment was conducted in accordance with the Laboratory Animals Service Center's guidelines.

### Cell culture

The prostate epithelial cells were enzymatically isolated from the rat prostate according to the method described by Shigeo Taketa [69] with some modifications. In brief, the ventral prostates of 4-week-old rats were removed and placed into a petri dish containing sterile HBSS. After washing with HBSS and the prostates were sliced into small pieces. The sliced tissue were placed in RIPM 1640 medium containing 0.1% collagenase I (Sigma) and incubated at 35°C for 45 min with constant shaking. After enzyme digestion, the supernatant fraction was passed through a 100-µm mesh cell strainer (BD Falcon™). The filtrate was centrifuged at 1000 rpm for 5 min. The tissue pieces remaining in the tube were subjected to digest twice as before and the final supernatant was pooled with the first fraction after sieving. The combined cells were collected by centrifugation at 1000 rpm for 5 min and then washed twice. The cells were finally suspended in RIPM 1640 medium containing 10% FBS and incubated at 37°C in a humidified atmosphere containing 5% CO_2_ overnight to remove fibroblasts. The cell supernatant was collected and cultured in a new culture flask with McCoy's 5A (Sigma) medium containing 5% FBS, 10 ug/mL Insulin (Sigma),10 ug/mL EGF (Sigma), 5 ug/ml transferring (Sigma), 10 ng/ml cholera toxin (Sigma), 25 ug/mL Bovine pituitary extract (BD), 100 U/ml penicillin, 100 µg/ml streptomycin for further study.

### Total RNA Extraction and Semi-quantitative Reverse Transcription PCR (RT-PCR)

Approximately 2×10^5^ cells/well were seeded in 6-well cell culture plates and grown to 80% confluence. Total RNA was extracted from cells using TRIzol reagent (Invitrogen) according to the manufacturer's suggestion. The RNA of *E.coli DH5*α in different concentration of bicarbonate and pH were extracted using Trizol Bacterial isolation kit *(*Invitrogen). Complementary DNA was synthesized from total RNA using M-MLV reverse transcriptase (USB, GE Healthcare). The resulting first strand cDNA sample was directly used for polymerase chain reaction (PCR). The sequences of primers and annealing temperature in PCR reaction were showed in Table 1.

**Table 1:** Primers and RT-PCR conditions.

Primer name		Sequence(5′→3′)	Length(bp)	Annealing temperature(°C)
GAPDH	ForwardReverse	GACCACAGTCCATGCCATCACTGC GCTGTTGAAGTCGCAGGAGACAAC	340	55
CFTR	ForwardReverse	AACTGAGACCTTACGCAG AGAAGCTCTGGTCCTCTG	481	55
CAII	ForwardReverse	ATGACCCTTCCCTACAGC GGTCACACATTCCAGCAG	503	56
IL-1β	ForwardReverse	CAACAAAAATGCCTCGTGC TGCTGATGTACCAGTTGGG	313	58
IL-6	ForwardReverse	AAATCTGCTCTGGTCTTCTGG TTAGATACCCATCGACAGG	414	55
TNF-α	ForwardReverse	TACTGAACTTCGGGGTGATCG CCT TGTCCCTTGAAGAGAACC	292	58
*E.coli* 16S	ForwardReverse	CTCCTACGGGAGGCAGCAGGWATTACCGCGGCKGCTG	162	55
*E.coli* IF1	ForwardReverse	ATGGCCAAAGAAGACAATATTG AGCGACTACGGAAGACAATG	216	55
*E.coli* IF2	ForwardReverse	TTTCCGCTTCAATCACTTTAC CCTTGCTGAAACTGTCTACTG	165	55
*E.coli* IF3	ForwardReverse	AAAGGCGGAAAACGAGTTC CCTTACTGTTTCTTCTTAGGAGCG	541	55

### Western Blotting

Cultured cells were lysed using radioimmunoprecipitation assay (RIPA) buffer. The whole cell extracts were cleared by centrifugation and total protein concentration was determined using Bradford protein assay system (Bio-Rad, USA). Denatured 40 µg protein samples were separated on 8% SDS-polyacrylamide gels and then transferred to nitrocellulose Hybond™ membrane (Amersham). After being blocked with 4% nonfat milk in Tris Buffered Saline-Tween 20 (TBST), the membrane was incubated with a polyclonal rabbit CFTR antibody (Alomone Labs), CAII antibody (Santa Cruz Biotechnology) and β-tubulin (Santa Cruz Biotechnology) followed by corresponding secondary antibodies. The protein bands were visualized using ECL reagents.

### Immunofluorescent Staining

Isolated primary prostate cells were grown on coverslips for immunofluorescent studies. Cells were washed with PBS and fixed in 4% paraformaldehyde (PFA) for 10 min. After permeabilized in 0.5% Triton X-100, cells was blocked by 10% normal goat serum for 30 min at room temperature, followed by CFTR antibody (Alomone Labs), cytokeratin 5 & 8 antibody (NeoMarkers from Research Diagnostics Inc) at 4°C overnight. Cells were washed with PBS and incubated with secondary antibody (Alex 488-conjugated IgG (Molecular Probes)) in dark room. Unbounded antibody was removed by washing and then counterstained with DAPI (Sigma). Finally, the slides were mounted to observe under Nikon eclipse 80i microscope with Nikon intensilight C-HGF1 Fluorenscence transmitter. Negative controls were performed by omission of primary antibodies and replacing it with PBS.

### Immunohistochemistry

SD rat prostate tissues and human prostate hyperplasia samples were cut and dried onto Superfrost microscope slides. After deparaffining and rehydrating, endogenous peroxidase activity was quenched using 3% hydrogen peroxide incubation for 30 min. Then the slides were placed in 10 mM sodium citrate buffer (pH 6) by boiling for antigen retrieval. The samples were blocked in 10% goat serum and then incubated overnight at 4°C with CFTR antibody(Alomone Labs) for rat tissues or CFTR antibody (Alexis Biochemicals) and CAII antibody (Santa Cruz Biotechnology) for human tissues. Visualization of specific interactions was monitored by using the UltraVision One HRP Polymer detection system (Thermo Fisher Scientific) according to the manufacture's instructions and the staining was visualized using DAB Plus Chromogen, followed by counterstained with hematoxylin. Negative controls were performed for tissue sections by using PBS.

### Measurement of intracellular pH in primary prostate epithelial cells

To establish a cell culture system of polarized prostate primary epithelial cells with both apical and basolateral compartment, the isolated cells were plated at a density of about 2×10^5^cells/ml onto Transwell-Col membranes with pores of 0.45 µm and pre-coated with Matrigel basement membrane matrix (1∶8 in PBS, BD Bioscience) floating on culture medium to reach confluence for 5–6 days. For intracellular pH (pHi) measurement, 5 µM 2′, 7′-bis-2(2-carbosyethyl)-5-(and-6)- carboxyfluorescence, acetoxymethyl ester (BCECF AM, Molecular Probes) was added. The fluorescence signal was recorded by a fluorescence microscope (Nikon Eclipse Ti). Using an excitation wavelength of 490/440 nm and an emission of 530 nm, a radiometric analysis of fluorescence data was performed using Metafluor software.

### *In vitro Escherichia coli* 055:B5 LPS stimulation

The stimulation experiment was performed in cultured epithelial cells. LPS from *E.coli* 055:B5 (Sigma) was used. Primary prostate epithelial cells were stimulated with 1 µg/ml LPS for 24 h.

### Antibacterial assay *in vitro*

The isolated cells were plated onto Transwell-Col membranes (0.45 cm^2^) coated with Matrigel and cultured like before. For infection, a total of 1×10^5^colony-forming units (CFU) of *E.coli DH5*α were added to the apical compartment of the epithelial cells. For blocking of CFTR or carbonic anhydrase, the *E coli* treated epithelial cells were preincubated with 10 µM CFTR_inh_-172 (Sigma), a specific CFTR channel blocker or anti-CFTR antibody (1∶500, Alomone labs) or 50 µM Acetazolamide (Sigma), a carbonic anhydrase inhibitor for 24 h before the addition of bacteria. Same concentration of DMSO or control IgG was added as control. After 18 h, the apical medium was collected for CFU counting on a Luria broth agar plate.

### Bacterial killing capacity *in vivo*

The 12-week-old male SD rats were injected 200 µl of 1×10^8^ CFU/ml *E.coli* suspension or combined with 10 µM CFTR_inh_-172 directly beneath the capsules of two sides of ventral lobes. We excluded two rats from the study which died of sepsis. The animals were killed 48 h after surgery and the ventral prostates from each rat were weighted, sliced into pieces and sonicated for 30 min in PBS for bacteria counting. The bacterial counts were expressed as the log of CFU per gram of prostate.

### Antibacterial assay in different concentration of bicarbonate and pH

*E.coli DH5*α was grown in a Luria broth at 37°C in a shaker to grow to log phase. Assay solutions were prepared as the Table 2 using NaCl to keep the same concentration of sodium in the solutions. The solutions were made without fixing the pH and allow the change of HCO_3_^−^ to alter its pH. The osmolarity of each solution was the same. A concentration of 1×10^9^ CFU/ml of *E.coli* was incubated at 37°C in the absence or presence of different concentration of NaHCO_3_ for 2 h and then OD_600_ was read. Another set of experiments was to examine the bactericidal activities of pH. A range of different pH values (7.35, 7.95, 8.14 and 8.24) in 25 mM HCO_3_^−^ which was corresponding with the pH value in different concentration of bicarbonate was achieved. *E.coli* was incubated for 2 h and OD_600_ was read.

**Table 2:** Solutions for different concentration of HCO_3_^−^.

HCO_3_^−^ conc.(mM)	NaCl(mM)	NaHCO_3_(mM)	KH_2_PO_4_(mM)	Na_2_HPO_4_(mM)	KCl(mM)	PH value
0	137	0	1.47	7.81	2.68	7.35
25	112	25				7.95
50	87	50				8.14
60	77	60				8.17
80	57	80				8.24

### Intracellular cAMP measurement

*E.coli* were exposed to different concentrations of HCO_3_^−^ in LB broth for 2 h and the cultures were harvested by centrifugation at 3,000×g for 10 min. The cAMP concentrations in *E.coli* were determined by using the Elisa kit (Assay Designs, Ann Arbor, MI) according to the manufacturer's instructions. The average intracellular cAMP concentration (determined in triplicate) was expressed in picomoles per milliliter (pmol/ml).

### Human prostate sample collection

Seven human prostate samples were obtained by transurethral resection from the suspected benign hyperplasia patients. Four samples from the patients with prostatitis showed evidence of lymphocytes infiltrates that were primarily centered in the peri-acinar region and stroma around acini and ducts. Tissue samples were obtained immediately after surgery and fixed in 4% paraformaldehyde (PFA) for paraffin section. Ethics approval for the study was obtained from the Ethics Committee of Peking University Shenzhen Hospital (Approval ID: 20090017) and all patients signed informed consent approving the use of their tissues for research purposes.

### Statistical Analyses

Data are expressed as the mean ± S.E.M. The two-tail Student's t tests were used to compare the difference between two groups. A probability of P<0.05 was considered to be statistically significant.

## References

[1] Snow DC, Shoskes DA (2010). Pharmacotherapy of prostatitis.. Expert Opin Pharmacother.

[2] Lipsky BA, Byren I, Hoey CT (2010). Treatment of bacterial prostatitis.. Clin Infect Dis.

[3] Giamarellou H, Tympanidis K, Bitos NA, Leonidas E, Daikos GK (1984). Infertility and chronic prostatitis.. Andrologia.

[4] Wolff H, Bezold G, Zebhauser M, Meurer M (1991). Impact of clinically silent inflammation on male genital tract organs as reflected by biochemical markers in semen.. J Androl.

[5] Leib Z, Bartoov B, Eltes F, Servadio C (1994). Reduced semen quality caused by chronic abacterial prostatitis: an enigma or reality?. Fertil Steril.

[6] Cunningham KA, Beagley KW (2008). Male genital tract chlamydial infection: implications for pathology and infertility.. Biol Reprod.

[7] Mitsumori K, Terai A, Yamamoto S, Ishitoya S, Yoshida O (1999). Virulence characteristics of Escherichia coli in acute bacterial prostatitis.. J Infect Dis.

[8] de la Rosette JJ, Hubregtse MR, Meuleman EJ, Stolk-Engelaar MV, Debruyne FM (1993). Diagnosis and treatment of 409 patients with prostatitis syndromes.. Urology.

[9] Alexander RB, Ponniah S, Hasday J, Hebel JR (1998). Elevated levels of proinflammatory cytokines in the semen of patients with chronic prostatitis/chronic pelvic pain syndrome.. Urology.

[10] Jang TL, Schaeffer AJ (2003). The role of cytokines in prostatitis.. World J Urol.

[11] He L, Wang Y, Long Z, Jiang C (2010). Clinical significance of IL-2, IL-10, and TNF-alpha in prostatic secretion of patients with chronic prostatitis.. Urology.

[12] Fair WR, Cordonnier JJ (1978). The pH of prostatic fluid: a reappraisal and therapeutic implications.. J Urol.

[13] Pfau A, Perlberg S, Shapira A (1978). The pH of the prostatic fluid in health and disease: implications of treatment in chronic bacterial prostatitis.. J Urol.

[14] Blacklock NJ, Beavis JP (1974). The response of prostatic fluid pH in inflammation.. Br J Urol.

[15] Weidner W (1992). Prostatitis–diagnostic criteria, classification of patients and recommendations for therapeutic trials.. Infection.

[16] White MA (1975). Change in pH of expressed prostatic secretion during the course of prostatitis.. Proc R Soc Med.

[17] Chen J, Xu Z, Zhao H, Jiang X (2007). Citrate in expressed prostatic secretions has the feasibility to be used as a useful indicator for the diagnosis of category IIIB prostatitis.. Urol Int.

[18] Thin RN (1991). The diagnosis of prostatitis: a review.. Genitourin Med.

[19] Chandiok S, Fisk PG, Riley VC (1992). Prostatitis–clinical and bacterial studies.. Int J STD AIDS.

[20] Winningham DG, Nemoy NJ, Stamey TA (1968). Diffusion of antibiotics from plasma into prostatic fluid.. Nature.

[21] Bjerklund Johansen TE, Gruneberg RN, Guibert J, Hofstetter A, Lobel B (1998). The role of antibiotics in the treatment of chronic prostatitis: a consensus statement.. Eur Urol.

[22] Hug MJ, Tamada T, Bridges RJ (2003). CFTR and bicarbonate secretion by [correction of to] epithelial cells.. News Physiol Sci.

[23] Strong TV, Boehm K, Collins FS (1994). Localization of cystic fibrosis transmembrane conductance regulator mRNA in the human gastrointestinal tract by in situ hybridization.. J Clin Invest.

[24] Cohn JA, Melhus O, Page LJ, Dittrich KL, Vigna SR (1991). CFTR: development of high- affinity antibodies and localization in sweat gland.. Biochem Biophys Res Commun.

[25] Chan HC, Shi QX, Zhou CX, Wang XF, Xu WM (2006). Critical role of CFTR in uterine bicarbonate secretion and the fertilizing capacity of sperm.. Mol Cell Endocrinol.

[26] Kopelman H, Corey M, Gaskin K, Durie P, Weizman Z (1988). Impaired chloride secretion, as well as bicarbonate secretion, underlies the fluid secretory defect in the cystic fibrosis pancreas.. Gastroenterology.

[27] Poulsen JH, Fischer H, Illek B, Machen TE (1994). Bicarbonate conductance and pH regulatory capability of cystic fibrosis transmembrane conductance regulator.. Proc Natl Acad Sci U S A.

[28] Lee MG, Choi JY, Luo X, Strickland E, Thomas PJ (1999). Cystic fibrosis transmembrane conductance regulator regulates luminal Cl-/HCO3- exchange in mouse submandibular and pancreatic ducts.. J Biol Chem.

[29] Ishiguro H, Steward M, Naruse S (2007). Cystic fibrosis transmembrane conductance regulator and SLC26 transporters in HCO3(-) secretion by pancreatic duct cells.. Sheng Li Xue Bao.

[30] Wang XF, Zhou CX, Shi QX, Yuan YY, Yu MK (2003). Involvement of CFTR in uterine bicarbonate secretion and the fertilizing capacity of sperm.. Nat Cell Biol.

[31] Chan HC, Ruan YC, He Q, Chen MH, Chen H (2009). The cystic fibrosis transmembrane conductance regulator in reproductive health and disease.. J Physiol.

[32] Chen MH, Chen H, Zhou Z, Ruan YC, Wong HY (2010). Involvement of CFTR in oviductal HCO3- secretion and its effect on soluble adenylate cyclase-dependent early embryo development.. Hum Reprod.

[33] Walker J, Watson J, Holmes C, Edelman A, Banting G (1995). Production and characterisation of monoclonal and polyclonal antibodies to different regions of the cystic fibrosis transmembrane conductance regulator (CFTR): detection of immunologically related proteins.. J Cell Sci.

[34] Hihnala S, Kujala M, Toppari J, Kere J, Holmberg C (2006). Expression of SLC26A3, CFTR and NHE3 in the human male reproductive tract: role in male subfertility caused by congenital chloride diarrhoea.. Mol Hum Reprod.

[35] Qiao D, Yi L, Hua L, Xu Z, Ding Y (2008). Cystic fibrosis transmembrane conductance regulator (CFTR) gene 5T allele may protect against prostate cancer: a case-control study in Chinese Han population.. J Cyst Fibros.

[36] Cafferata EG, Gonzalez-Guerrico AM, Giordano L, Pivetta OH, Santa-Coloma TA (2000). Interleukin-1beta regulates CFTR expression in human intestinal T84 cells.. Biochim Biophys Acta.

[37] Ajonuma LC, He Q, Sheung Chan PK, Yu Ng EH, Fok KL (2008). Involvement of cystic fibrosis transmembrane conductance regulator in infection-induced edema.. Cell Biol Int.

[38] Choi JY, Muallem D, Kiselyov K, Lee MG, Thomas PJ (2001). Aberrant CFTR-dependent HCO3- transport in mutations associated with cystic fibrosis.. Nature.

[39] Quinton PM (2001). The neglected ion: HCO3.. Nat Med.

[40] Tate S, MacGregor G, Davis M, Innes JA, Greening AP (2002). Airways in cystic fibrosis are acidified: detection by exhaled breath condensate.. Thorax.

[41] Coakley RD, Grubb BR, Paradiso AM, Gatzy JT, Johnson LG (2003). Abnormal surface liquid pH regulation by cultured cystic fibrosis bronchial epithelium.. Proc Natl Acad Sci U S A.

[42] Thompson KD, Welykyj S, Massa MC (1993). Antibacterial activity of lidocaine in combination with a bicarbonate buffer.. J Dermatol Surg Oncol.

[43] Drake DR, Vargas K, Cardenzana A, Srikantha R (1995). Enhanced bactericidal activity of Arm and Hammer Dental Care.. Am J Dent.

[44] Craig SB, Concannon MJ, McDonald GA, Puckett CL (1999). The antibacterial effects of tumescent liposuction fluid.. Plast Reconstr Surg.

[45] Jarvis GN, Fields MW, Adamovich DA, Arthurs CE, Russell JB (2001). The mechanism of carbonate killing of Escherichia coli.. Lett Appl Microbiol.

[46] Dorschner RA, Lopez-Garcia B, Peschel A, Kraus D, Morikawa K (2006). The mammalian ionic environment dictates microbial susceptibility to antimicrobial defense peptides.. FASEB J.

[47] Bhattacharya A, Datta A (1977). Effect of cyclic AMP on RNA and protein synthesis in Candida albicans.. Biochem Biophys Res Commun.

[48] Kaul R, Tao S, Wenman WM (1990). Cyclic AMP inhibits protein synthesis in Chlamydia trachomatis at a transcriptional level.. Biochim Biophys Acta.

[49] Chen Y, Cann MJ, Litvin TN, Iourgenko V, Sinclair ML (2000). Soluble adenylyl cyclase as an evolutionarily conserved bicarbonate sensor.. Science.

[50] Hoglund P, Haila S, Socha J, Tomaszewski L, Saarialho-Kere U (1996). Mutations of the Down-regulated in adenoma (DRA) gene cause congenital chloride diarrhoea.. Nat Genet.

[51] Shcheynikov N, Wang Y, Park M, Ko SB, Dorwart M (2006). Coupling modes and stoichiometry of Cl-/HCO3- exchange by slc26a3 and slc26a6.. J Gen Physiol.

[52] Tajima Y, Okamura N, Sugita Y (1987). The activating effects of bicarbonate on sperm motility and respiration at ejaculation.. Biochim Biophys Acta.

[53] Zhou CX, Wang XF, Chan HC (2005). Bicarbonate secretion by the female reproductive tract and its impact on sperm fertilizing capacity.. Sheng Li Xue Bao.

[54] He Q, Tsang LL, Ajonuma LC, Chan HC (2010). Abnormally up-regulated cystic fibrosis transmembrane conductance regulator expression and uterine fluid accumulation contribute to Chlamydia trachomatis-induced female infertility.. Fertil Steril.

[55] Brouillard F, Bouthier M, Leclerc T, Clement A, Baudouin-Legros M (2001). NF-kappa B mediates up-regulation of CFTR gene expression in Calu-3 cells by interleukin-1beta.. J Biol Chem.

[56] Wagenlehner FM, Weidner W, Sorgel F, Naber KG (2005). The role of antibiotics in chronic bacterial prostatitis.. Int J Antimicrob Agents.

[57] Gualerzi CO, Pon CL (1990). Initiation of mRNA translation in prokaryotes.. Biochemistry.

[58] Grill S, Moll I, Hasenohrl D, Gualerzi CO, Blasi U (2001). Modulation of ribosomal recruitment to 5′-terminal start codons by translation initiation factors IF2 and IF3.. FEBS Lett.

[59] Cummings HS, Hershey JW (1994). Translation initiation factor IF1 is essential for cell viability in Escherichia coli.. J Bacteriol.

[60] Beeler JA, Tang WJ (2004). Expression and purification of soluble adenylyl cyclase from Escherichia coli.. Methods Mol Biol.

[61] Roelofs J, Van Haastert PJ (2002). Deducing the origin of soluble adenylyl cyclase, a gene lost in multiple lineages.. Mol Biol Evol.

[62] Xie F, Garcia MA, Carlson AE, Schuh SM, Babcock DF (2006). Soluble adenylyl cyclase (sAC) is indispensable for sperm function and fertilization.. Dev Biol.

[63] Sayner SL, Alexeyev M, Dessauer CW, Stevens T (2006). Soluble adenylyl cyclase reveals the significance of cAMP compartmentation on pulmonary microvascular endothelial cell barrier.. Circ Res.

[64] Corral LG, Post LS, Montville TJ (1988). Antimicrobial activity of sodium bicarbonate.. J food Sci.

[65] Schaeffer AJ, Wendel EF, Dunn JK, Grayhack JT (1981). Prevalence and significance of prostatic inflammation.. J Urol.

[66] Erdogan S, Aslantas O, Celik S, Atik E (2008). The effects of increased cAMP content on inflammation, oxidative stress and PDE4 transcripts during Brucella melitensis infection.. Res Vet Sci.

[67] Houslay MD, Adams DR (2003). PDE4 cAMP phosphodiesterases: modular enzymes that orchestrate signalling cross-talk, desensitization and compartmentalization.. Biochem J.

[68] Trevani AS, Andonegui G, Giordano M, Lopez DH, Gamberale R (1999). Extracellular acidification induces human neutrophil activation.. J Immunol.

[69] Taketa S, Nishi N, Takasuga H, Okutani T, Takenaka I (1990). Differences in growth requirements between epithelial and stromal cells derived from rat ventral prostate in serum-free primary culture.. Prostate.

